# Multilevel Lumbar Epidural Angiolipoma: A Case Report

**DOI:** 10.7759/cureus.117695

**Published:** 2026-10-09

**Authors:** Raphael Zimmermann, Sebastião Carlos Duarte, Ana Maria Adami, Marcos Garcia Lourenção, Allan Zimmermann

**Affiliations:** 1 Neurosurgery, Hospital São Vicente de Paulo, Jundiaí, BRA; 2 Neurosurgery, Hospital Paulo Sacramento, Jundiaí, BRA; 3 Pathology, Instituto Cardoso de Almeida Patologia, Jundiaí, BRA; 4 Neurological Surgery, Hospital São Vicente de Paulo, Jundiaí, BRA

**Keywords:** epidural tumor, foraminal extension, lumbar spine, magnetic resonance imaging, spinal angiolipoma, spinal neoplasm

## Abstract

Spinal angiolipomas are rare benign tumors composed of mature adipose tissue and vascular elements, most commonly located in the thoracic epidural space. Lumbar involvement is considerably less frequent, and variable proportions of adipose and vascular tissue may result in atypical magnetic resonance imaging (MRI) characteristics and diagnostic uncertainty. We report the case of a 36-year-old woman with an approximately one-year history of persistent low back pain associated with subjective bilateral lower-extremity weakness despite a preserved neurological examination. An initial non-contrast lumbar MRI was interpreted as showing no significant abnormality other than minimal degenerative changes; however, retrospective review revealed a subtle posterior epidural lesion. Because of persistent symptoms, further investigation with contrast-enhanced MRI was performed, demonstrating an elongated epidural lesion extending from L1 to L3, predominantly involving the posterior and right posterolateral epidural space with associated foraminal extension. The lesion demonstrated areas of low T1 signal intensity and intense homogeneous contrast enhancement. The patient underwent L1-L3 laminoplasty and gross-total resection. Intraoperatively, the lesion had a macroscopically adipose appearance and was moderately vascular. Histopathological examination demonstrated mature adipose tissue admixed with numerous vascular channels, confirming the diagnosis of spinal angiolipoma. Postoperatively, pain and subjective lower-extremity weakness improved without new neurological deficits, and early postoperative MRI showed no evidence of residual or recurrent tumor. This case highlights the variable imaging appearance of lumbar spinal angiolipomas and the value of correlating MRI characteristics with intraoperative and histopathological findings, particularly in enhancing epidural lesions without a predominantly fatty appearance.

## Introduction

Spinal angiolipomas are rare benign tumors composed of mature adipose tissue and abnormal vascular elements. They predominantly arise in the epidural space and account for approximately 0.04-1.2% of all spinal tumors and 2-3% of extradural spinal neoplasms [1,2]. Although they may occur throughout the spinal canal, the thoracic spine is by far the most frequently involved region, whereas lumbar localization is considerably less common [1-3].

Clinical manifestations are generally related to compression of neural structures and most commonly include back pain, sensory disturbances, and progressive motor weakness [1,2]. Magnetic resonance imaging (MRI) is the primary imaging modality for diagnosis; however, the radiological appearance may vary according to the relative proportions of adipose and vascular components [3,4]. Fat-predominant regions typically demonstrate high signal intensity on T1-weighted sequences, whereas areas with greater vascular content may demonstrate lower T1 signal intensity and prominent contrast enhancement [4,5].

We report a case of a multilevel lumbar epidural angiolipoma extending from L1 to L3 in a 36-year-old woman with persistent low back pain and subjective lower-extremity weakness despite the absence of objective neurological deficits. The lesion was not described in the initial MRI report and was subsequently characterized on contrast-enhanced imaging before surgical resection and histopathological confirmation. This case highlights the diagnostic challenge posed by lumbar spinal angiolipomas, particularly when the lesion lacks a predominantly fatty MRI appearance and presents with subtle findings on initial non-contrast imaging and associated foraminal extension.

## Case presentation

Clinical presentation

A 36-year-old woman was referred to our neurosurgical service with an approximately one-year history of progressive low back pain associated with a subjective sensation of weakness in both lower extremities. She reported no radicular pain, sensory disturbances, or bowel or bladder dysfunction. She had sought emergency medical care on multiple occasions because of the progressive and persistent symptoms. Neurological examination revealed preserved deep tendon reflexes, no objective motor deficit, and no pyramidal signs.

Radiological findings

Initial non-contrast lumbar magnetic resonance imaging (MRI) performed in April 2025 was interpreted as demonstrating only a minimal concentric posterior disc bulge at L5-S1. The spinal canal and conus medullaris were reported as normal.

Despite the absence of a significant abnormality in the initial radiological report, the patient’s symptoms persisted. Retrospective review of the initial MRI demonstrated a subtle posterior epidural lesion at the L1-L2 level (Figure 1), prompting further investigation with contrast-enhanced MRI.

**Figure 1:**
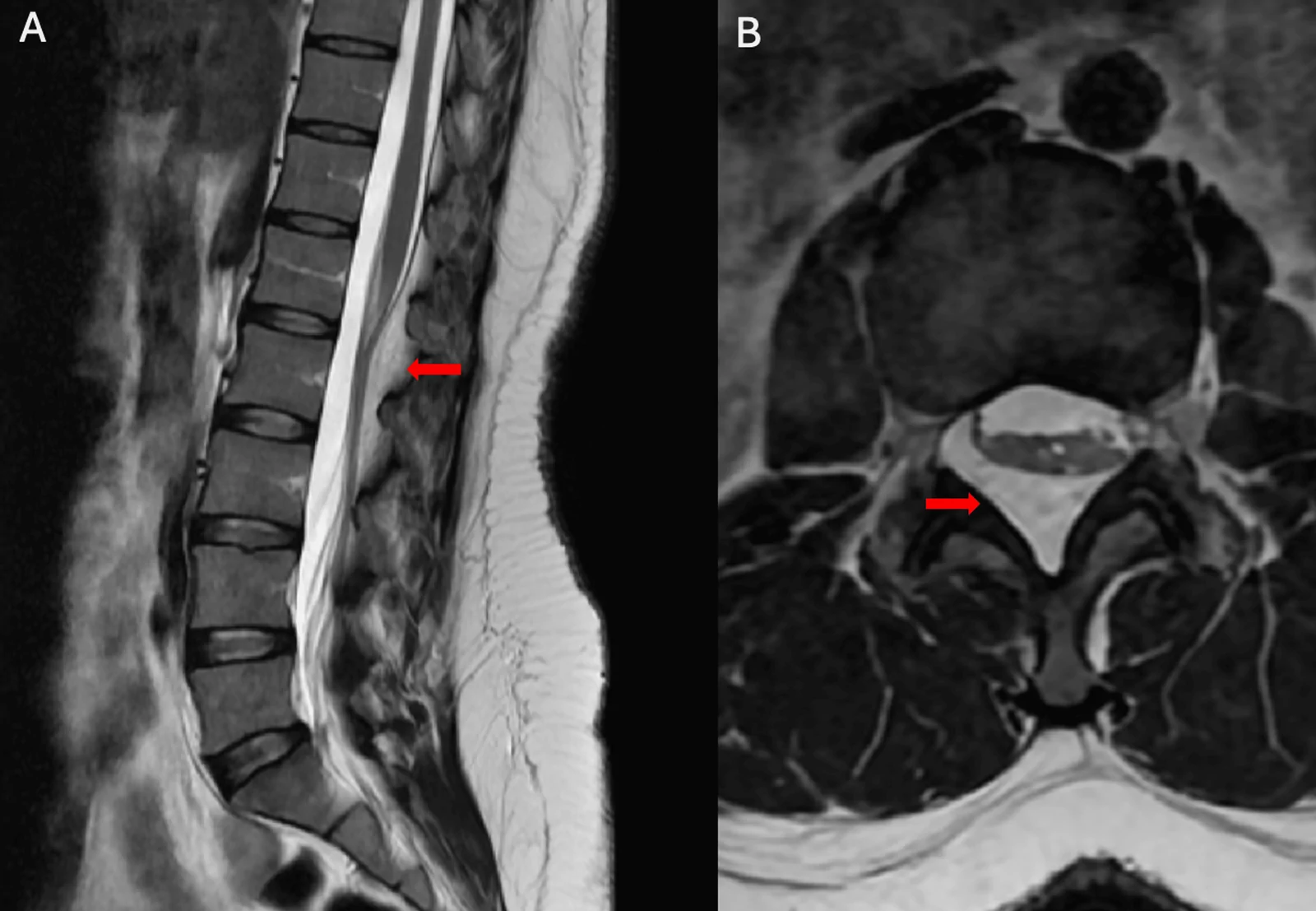
Initial non-contrast lumbar magnetic resonance imaging. Sagittal (A) and axial (B) T2-weighted magnetic resonance images obtained in April 2025. At the L1-L2 level, a retrospective review demonstrated a subtle posterior epidural lesion (red arrows), more appreciable on the axial image (B), which was not described in the initial radiological report.

Contrast-enhanced lumbar MRI performed in October 2025 demonstrated an elongated, fusiform epidural lesion extending from L1 to L3, predominantly involving the posterior and right posterolateral epidural space with associated foraminal extension (Figure 2). The lesion measured 5.4 × 1.7 × 0.5 cm and demonstrated heterogeneous hyperintensity on T2-weighted and STIR sequences, areas of low signal intensity on T1-weighted imaging, and intense homogeneous enhancement following contrast administration. The intraspinal component contacted the posterolateral aspect of the conus medullaris and adjacent nerve roots.

**Figure 2:**
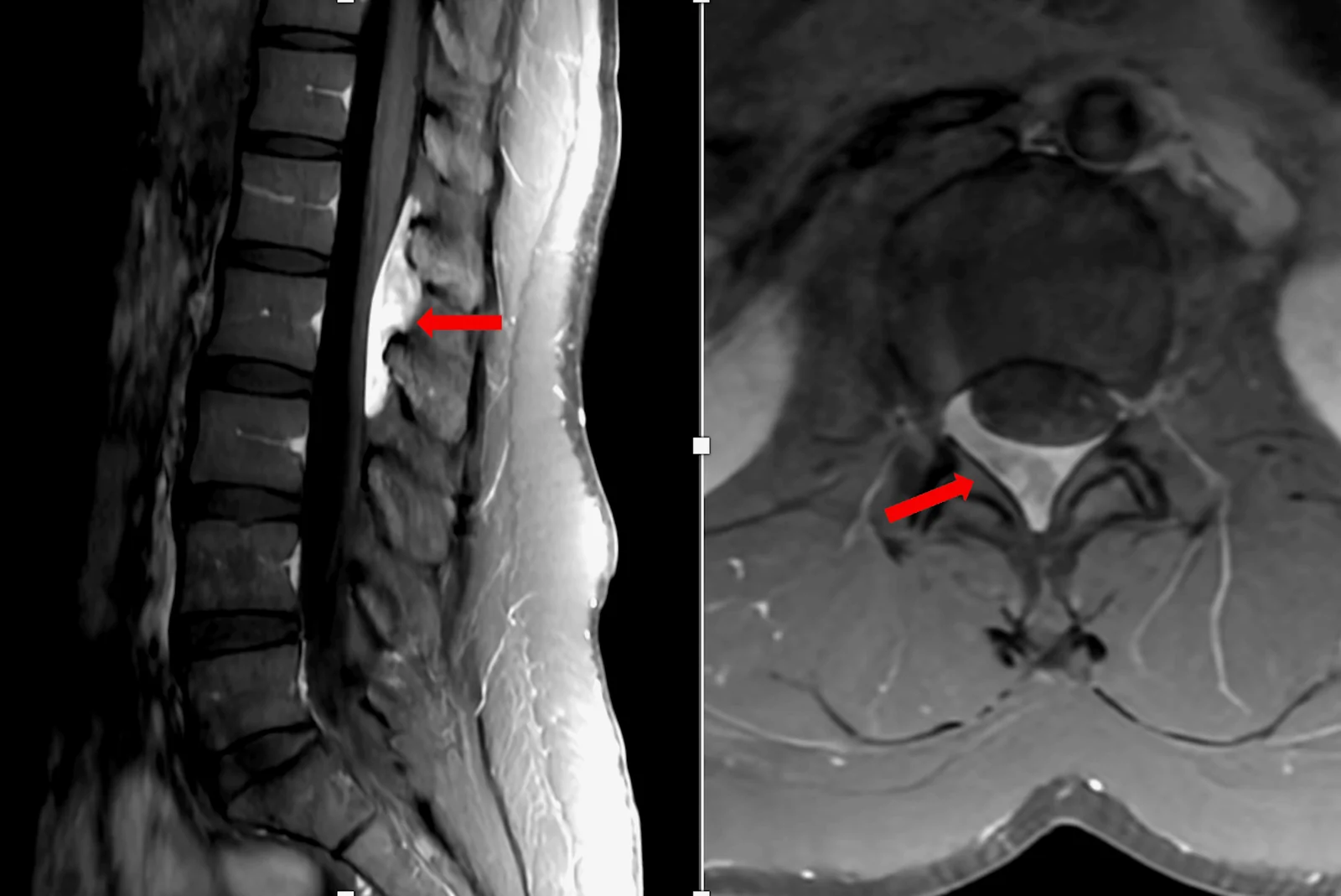
Contrast-enhanced magnetic resonance imaging of the lumbar epidural lesion. Sagittal (A) and axial (B) post-contrast T1-weighted images demonstrate the enhancing epidural lesion extending from L1 to L3. At the L1-L2 level, the lesion predominantly involves the posterior and right posterolateral epidural space, with associated foraminal extension. Red arrows indicate the lesion.

The radiological differential diagnosis included a meningothelial lesion, particularly meningioma, and a vascular lesion such as hemangioma.

Surgical treatment and histopathological findings

Given the persistence of symptoms and the presence of an epidural lesion contacting the conus medullaris and adjacent neural structures, surgical treatment was undertaken. In February 2026, the patient underwent L1-L3 laminoplasty. Intraoperatively, an extradural lesion with a macroscopically adipose appearance was identified. The lesion was moderately vascular, with moderate bleeding encountered during resection. Gross-total resection was considered to have been achieved based on intraoperative assessment.

Macroscopic pathological examination demonstrated an irregular 4.0 × 2.0 cm elastic, brownish tissue fragment partially covered by adipose tissue. Histopathological examination demonstrated mature adipose tissue admixed with numerous vascular channels, establishing the diagnosis of an extradural spinal angiolipoma (Figure 3). No evidence of malignancy was identified.

**Figure 3:**
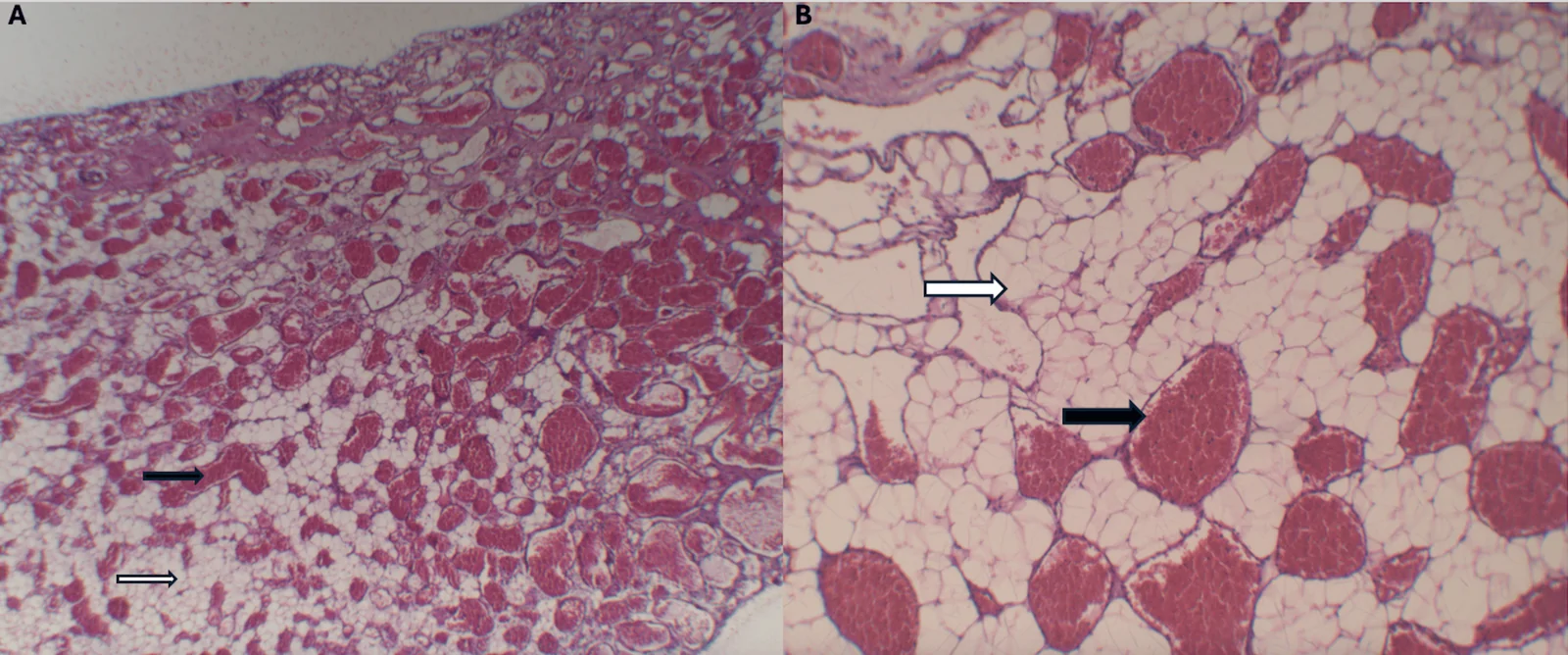
Histopathological findings of the spinal epidural angiolipoma. Representative hematoxylin and eosin (H&E)-stained sections at lower (A) and higher (B) magnification demonstrate the characteristic admixture of mature adipose tissue and numerous vascular channels, consistent with angiolipoma. White arrows indicate mature adipose tissue, and black arrows indicate vascular channels in both panels.

Postoperative course and follow-up

The patient experienced improvement in both pain and the subjective sensation of lower-extremity weakness after surgery, without new neurological deficits.

Follow-up contrast-enhanced lumbar MRI obtained in May 2026 demonstrated postoperative changes involving the posterior elements of L1-L3 and epidural postoperative tissue enhancement, without evidence of residual or recurrent tumor on early postoperative imaging (Figure 4).

**Figure 4:**
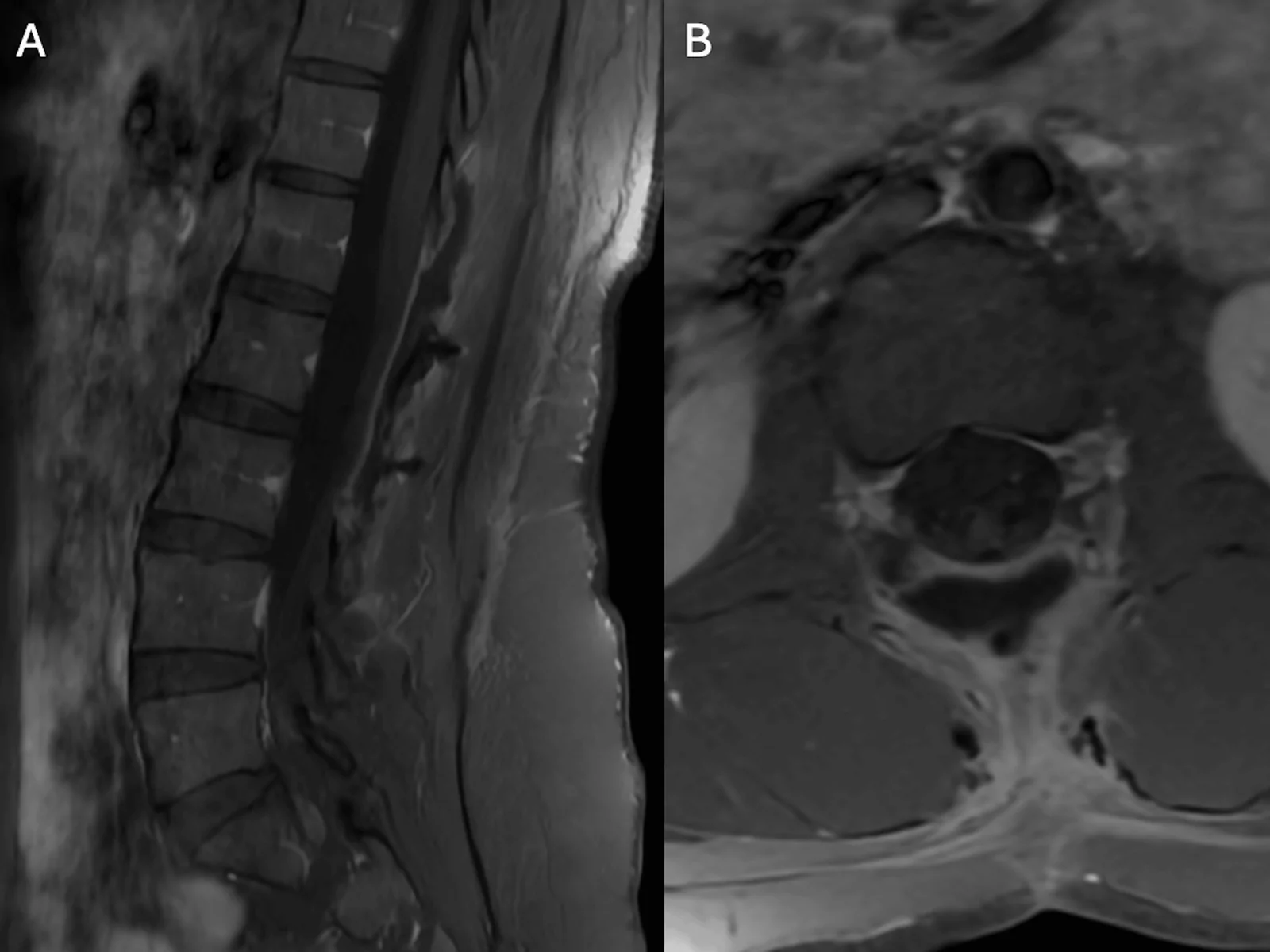
Postoperative contrast-enhanced lumbar magnetic resonance imaging. Sagittal (A) and axial (B) post-contrast T1-weighted magnetic resonance images obtained in May 2026 demonstrate postoperative changes following L1-L3 laminoplasty, with enhancement of the postoperative epidural tissues and no evidence of residual or recurrent tumor on early postoperative imaging.

## Discussion

Spinal angiolipomas are uncommon benign tumors with a marked predilection for the thoracic epidural space. In a recent systematic review by Yayla et al., which included 240 histologically confirmed cases, 72.9% were located in the thoracic spine, making lumbar involvement considerably less common [1]. Purely lumbar angiolipomas have therefore been reported mainly as isolated cases and small series [6-8]. In our patient, the lesion was entirely lumbar, extending from L1 to L3 and predominantly involving the posterior and right posterolateral epidural space with associated foraminal extension.

Clinical manifestations depend primarily on the location and degree of neural compression. Motor weakness and pain were reported in 55.4% and 42.9% of patients, respectively, in a systematic review by Yayla et al., with a median symptom duration of 12 months [1]. Nevertheless, lumbar angiolipomas may remain neurologically subtle. Dogan et al. described two patients with lumbar extradural angiolipomas presenting with low back pain without neurological signs [8], while Jammal et al. recently reported a lumbar epidural lesion associated with prolonged low back pain but no motor or sphincter deficits [7]. Similarly, our patient experienced approximately one year of persistent low back pain and subjective bilateral lower-extremity weakness despite a preserved objective neurological examination. These cases demonstrate that the absence of neurological deficits does not exclude a clinically relevant lumbar epidural lesion.

MRI findings in spinal angiolipomas are strongly influenced by the relative proportions of mature adipose tissue and vascular elements [3-5]. Fat-rich portions characteristically demonstrate high signal intensity on T1-weighted imaging, whereas more vascular regions may exhibit relatively lower T1 signal and prominent enhancement after gadolinium administration [3-5]. Hu et al. demonstrated that T1-hypointense areas correspond to more vascular portions of these tumors [4], consistent with earlier observations by Provenzale and McLendon [5]. In our patient, the lesion was retrospectively visible on the initial non-contrast MRI but was not described in the original radiological report. On the subsequent contrast-enhanced examination, obtained approximately six months later, the lesion was substantially more conspicuous and demonstrated areas of low T1 signal intensity with intense enhancement. Because approximately six months elapsed between examinations, the increased conspicuity cannot be attributed solely to contrast administration. The margins of the lesion on the initial non-contrast examination were insufficiently defined to permit a reliable retrospective comparison of dimensions; therefore, interval tumor growth cannot be excluded as a contributing factor. The prominent enhancement correlated with the moderate intraoperative vascularity and the numerous vascular channels identified histopathologically, providing radiologic-surgical-pathologic correlation.

The variable proportions of adipose and vascular tissue may also broaden the preoperative differential diagnosis. Spinal angiolipomas can resemble other enhancing epidural lesions, including meningiomas, vascular lesions, lymphomas, and neurogenic tumors [3,9,10]. In our patient, the preoperative differential diagnosis included meningioma and hemangioma. Foraminal involvement provided an additional diagnostic consideration. Akyuva et al. reported a posterolateral lumbar angiolipoma with neural foraminal expansion that radiologically mimicked a schwannoma [10]. Although foraminal extension was also present in our patient, the lesion had a broader posterior and posterolateral epidural component rather than a predominantly foraminal configuration. Thus, the absence of a predominantly fatty appearance should not exclude angiolipoma from the differential diagnosis of an enhancing lumbar epidural mass.

Surgical resection remains the treatment of choice for symptomatic spinal angiolipomas and is generally associated with favorable outcomes [1,2,11]. Although infiltrating lesions involving adjacent structures may make complete removal more difficult [1,11], recurrence is uncommon; the recent systematic review reported recurrence in only 0.8% of cases, while acknowledging limitations related to heterogeneous follow-up [1]. Favorable postoperative outcomes have also been reported specifically for lumbar lesions [7,12]. Our patient underwent L1-L3 laminoplasty with gross-total resection based on intraoperative assessment, followed by improvement in pain and subjective lower-extremity weakness without new neurological deficits. Early postoperative MRI demonstrated no evidence of residual or recurrent tumor. However, given the short follow-up interval of approximately two months, longer-term imaging surveillance is necessary to assess recurrence more definitively.

This case emphasizes that lumbar epidural angiolipoma should remain in the differential diagnosis of enhancing epidural masses, particularly when persistent symptoms are associated with subtle non-contrast findings and prominent enhancement. The correlation between MRI characteristics, intraoperative vascularity, and histopathological composition may be particularly useful in understanding these diagnostically challenging presentations.

## Conclusions

Spinal angiolipoma should be considered in the differential diagnosis of lumbar epidural masses even when the characteristic appearance of a predominantly fatty lesion is absent. Variability in the relative proportions of adipose and vascular tissue may substantially influence MRI characteristics, with vascular-rich components demonstrating lower T1 signal intensity and prominent contrast enhancement. In the present case, the correlation between intense enhancement, intraoperative vascularity, and histological vascular channels was particularly informative. Surgical resection resulted in symptomatic improvement without neurological deterioration, with no evidence of residual or recurrent tumor on early postoperative imaging. Longer-term follow-up is necessary to assess sustained tumor control.
